# Dental Treatment Needs of Male Inmates in Relation to the Analysis of Medical Databases

**DOI:** 10.3390/jcm13030858

**Published:** 2024-02-01

**Authors:** Rafał Korkosz, Agata Trzcionka, Dagmara Mączkowiak, Maksymilian Kiełbratowski, Anna Kuśka-Kiełbratowska, Mansur Rahnama, Marta Tanasiewicz

**Affiliations:** 1 Department of Conservative Dentistry with Endodontics, Faculty of Medical Sciences in Zabrze, Medical University of Silesia, Plac Akademicki 17, 41-902 Bytom, Poland; rkorkosz@sum.edu.pl (R.K.); dagmara.maczkowiak@gmail.com (D.M.); mkielbratowski@sum.edu.pl (M.K.); martatanasiewicz@sum.edu.pl (M.T.); 2 Department of Periodontal Diseases and Oral Mucosa Diseases, Faculty of Medical Sciences in Zabrze, Medical University of Silesia, pl. Traugutta 2, 41-800 Zabrze, Poland; 3 Department of Dental Surgery, Medical University of Lublin, Doktora Witolda Chodźki 6, 20-093 Lublin, Poland; mansur.rahnama@umlub.pl

**Keywords:** oral status, oral hygiene, prisoners, inmates, treatment needs, endodontic treatment

## Abstract

The worldwide incarceration rate per 100,000 people varies from 30 in India, 580 in Europe, to 750 in United States. The health of prisoners is of great concern. Research in many countries has shown poor oral health conditions among prisoners, particularly reflected in the high number of lost and untreated decayed teeth. The aim of our study was to evaluate the quality and range in dental procedures conducted on male prisoners, based on a retrospective analysis of medical history gathered at the Academic Center of Dentistry and Specialistic Medicine of Medical University of Silesia in Bytom for the period 2018–2021, and its correlation with the results obtained from the analysis of the Web of Science (WoS) and SCOPUS medical databases. Our research was carried out on the examined group, 86 men (mean age 31 years old), and a control group, 106 men (mean age 32 years old). The retrospective analysis of the medical history was performed. Results of our own research showed the values for decayed and missing teeth were significantly higher in the examined group while the values for the filled teeth component were significantly higher in the control group. The chance for the occurrence of the radices was 2.5 times higher in the examined group than in the control. The examined group was characterized by 3.6 times higher chance of no root canal treatment than the control group. The number of the endodontically treated teeth was significantly higher in the control group. The examined group was characterized by 4.2 higher probability for periapical lesion occurrence in teeth that were not endodontically treated. The number of teeth qualified for the endodontic treatment was significantly higher in the examined group, while the number of teeth qualified for the reendow treatment was significantly higher in the control group. The probability for the occurrence of both vertical and horizontal atrophy in the alveolar process was twice and three times higher in the examined than in the control group. In conclusion, the oral status of inmates is worse when compared to those who live in freedom, which is why there is a need to prepare a scheme to improve the condition of the stomatognathic system in prisoners.

## 1. Introduction

The values of global incarceration rate per 100,000 people ranges from 9 in San Marino, 36 in Nigeria, 40 in India, 106 in European Union, 250 in South Africa, 531 in Unites States, to 1086 in El Salvador [1]. In Poland, this indicator is estimated to be at 208 [1].

The health of prisoners is of great concern, particularly because the numbers of people under the jurisdiction of correction system continues to increase dramatically [2]. Prisoners usually come from disadvantaged groups with less education, higher unemployment rates, and lower chances for adopting healthy practices and having access to and seeking health care [2].

According to written records, the state of prisoners’ oral health is strongly connected to their frequent use of substances such as tobacco, alcohol, and illicit drugs [2,3,4,5,6,7,8]. Research in many countries has shown poor oral health condition among prisoners, particularly reflected in the high number of lost and untreated decayed teeth [2,3,4,5,6,7,8,9,10,11]. While serving their sentences, inmates may develop even worse and deficient health conditions associated with not only their stay in the penitentiary system, but also with their unwillingness to have a healthy lifestyle and to comply with generally accepted oral hygiene standards [7].

Many challenges exist in delivering services in the prison system, including a service provision with respect to security procedures, recruitment, and retention of dental staff in relation to strong demand and lucrative remuneration for dentists in private practice [2]. 

To this end, some countries have implemented guidelines that include improving dental health care for inmates, such as Brazil’s National Plan of Prison Health [4]. Living up to the needs of the inmates’ oral cavities health is not characteristic just for the developing countries. 

Finnish scientists have also confirmed their hypothesis concerning a much worse state of oral health of inmates stationed at the Pelso Prison in relation to the whole population of mature Finns [6]. Staying in prison in any country is linked with restricted access to dental care in the place of imprisonment. The inmates in Poland are able to access professional dental care outside their place of confinement. They are then escorted by prison guards to a medical center and not charged for any of the medical procedures. Very often they manifest their willingness for dental extraction instead of multiple-visit endodontic treatment [7].

Brazilians have noticed a relationship between the DMFT in the 22–32 range and the lack of inmate satisfaction concerning their state of the oral cavity, which was closely tied to their trouble with speech and prominent embarrassment [4]. Among inmates of the Russian Federation, 92.4% having prevalence caries and only 10.5% presented all teeth [7]. The mean DMFT index for the examined group of inmates in the Russian Federation was 14.86 [7]. Amongst the Finnish inmates, average DMFT was estimated at 16.8%, but none were observed to lack teeth [6].

In Poland, inmates are considered as a vulnerable group with special health needs. Improving the oral health of prison inmates is a challenging task [5]. 

The aim of our study is to evaluate the quality and range in dental procedures conducted on male prisoners, based on retrospective analysis of medical history gathered at the Academic Center of Dentistry and Specialistic Medicine of Medical University of Silesia in Bytom for the period 2018–2021. 

## 2. Materials and Methods

The retrospective study of medical history of patients treated in the Academic Center of Dentistry and Specialistic Medicine (ACDaSM) of Medical University of Silesia in Bytom (for the period 2018–2021) was conducted. The agreement from the personal data controller for their anonymous usage was obtained based on compliance with the retrospective study. 

The examined group (EG) was composed of 86 imprisoned men (prisoners from the Upper Silesia, average age 31.0) who were patients of ACDaSM during 2018–2021. The control group (CG) was composed of 106 randomly chosen men (average age 32.0) who were treated at ACDaSM during 2018–2021. 

The retrospective analysis of medical history was performer in order to receive data regarding:

- Patients’ age;
- DMFT (decayed–missing–filled teeth) index

The index is a sum of the number of decayed (D—a tooth diagnosed with one or more cavities, neither primary or secondary), missing (M—a tooth lost because of caries), and filled (F—a tooth with one or more fillings, with a prosthetic crown but with no secondary caries),

- Number of remaining roots;
- Number of endodontically treated teeth assessed using the following indices for the quality of the root canal treatment:

Radiological assessment of teeth due to *De Cleena* with *Bołtacz-Rzepkowska* modification. The tooth is regarded to be endodontically treated if its chamber and/or roots were obturated with the usage of contrast material. The quality of root canal treatment is evaluated in a binary system: proper only when the root canal obturation ends 0–2 mm from the root apex; improper when the root canal obturation ends higher than 2 mm from the root apex, the root canal is over-obturated, the root filling material is pushed into the periapical tissues, or in the case of lacking obturation homogeneity [12,13].

Radiological assessment by Strindberg reconsiders three possible outcomes of the root canal therapy: positive—when the width and the outline of the periodontal gap is proper or when the periodontal gap is slightly enlarged around the excessive filling material; unsure—when the results are questionable; and negative—failure, when the presence of periapical lesions is diagnosed [13].

Periapical tissue condition with the usage of periapical index (PAI) by Ostravik: Grade 1—normal periapical structures; 

Grade 2—small changes in bone structure; 

Grade 3—structural bone changes with some mineral loss;

Grade 4—periodontitis with well-defined radiolucency; 

Grade 5—apical radiolucency with bone expansion.

This index is of crucial importance when assessing root canal treatment effectiveness for teeth with periapical lesions, as it provides the possibility for monitoring the healing process on the basis of X-rays taken during recalls [13,14]:

- Assessment of periodontium status in endodontically treated teeth;
- Number of teeth qualified for primary or secondary root canal treatment;
- Assessment of the marginal periodontal tissues condition on the basis of vertical or horizontal bone resorption presence visible with X-ray analysis.

The description of the digital panoramic X-rays was performed by a dentist who had acquired certification through a dental radiology course. Roentgenogram assessment took place at a site equipped with a DICOM monitor and in a room with matt walls, where no reflective objects were present. The X-rays were compared to the data featured in the patient’s medical history. Information about the mode (emergency/planned) and the purpose of the appointment (according to code ICD-10 from the International Classification of Diseases) was obtained. Data were gathered concerning the conservative dentistry, surgery, and dental prosthetics procedures. 

Inclusion criteria included the medical history of adult men treated in ACDaSM during the period 2018–2021 and having baseline X-rays. Exclusion criteria were medical histories with no baseline panoramic X-rays, medical histories of patients under 18, women, and medical histories from years other than 2018–2021.

### Statistical Analysis

To analyze the data obtained, a comparison of both groups (examined and control) was performed. The quantitative data were analyzed with the *Mann–Whitney* test and for the qualitative chi-square test for two independent samples. For cases when the qualitative variable was dichotomous, the odds ratio (OR) with a 95% confidence interval was calculated. The odds ratio was verified with the *Mantel–Haenszel* test. The sensitivity and specificity values were also calculated. In particular cases, the results of the chi-square tests were complemented with the *Fisher exact* test. Excel and Statistica 2010 were used to carry out the analysis. 

## 3. Results

The average age of the 86 men who qualified for the examined group was 31.00 years. There was no statistically significant difference between the examined group and the control group (106 men, average age 32.00) was observed. The Mann–Whitney test was used in order to correlate the age (*p* = 0.227). 

**Figure 1:**
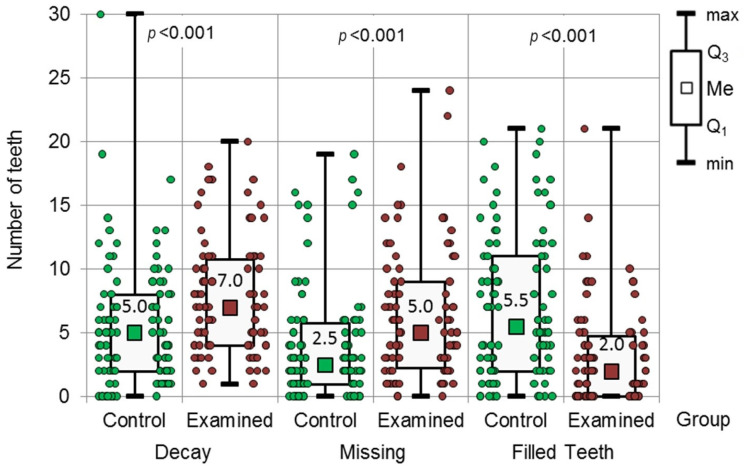
Results of the Mann–Whitney test regarding the DMF index.

The DMF index value for the examined group was 17.50 and 16.00 for the control group; no statistically significant differences were observed (*p* = 0.207, Mann–Whitney test). It was observed, however, that the D and M values were significantly higher in the examined group (*p* < 0.001). The values were D—7.0 for the examined group and 5.0 for the control group, M—5.0 in the examined group and 2.5 in the control group. The value for F was significantly higher in the control group, 5.5 (*p* < 0.001), in comparison to the examined group, 2.0. The values for particular components of the DMF index are presented in Figure 1.

**Figure 2:**
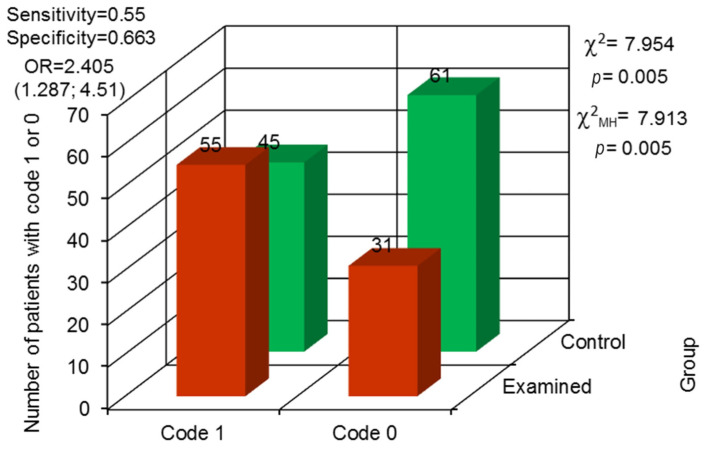
Odds ratio (OR) with 95% confidence interval and assessment for the variability in differences between both groups concerning retained roots (code 1—observed, code 0—not observed).

Retained roots were observed more often in the examined group; they were present in 55 patients and not observed in 31. In the control group, retained roots were noticed in 45 patients and in 61 they were not. The odds ratio proved that the examined group was characterized by almost 2.5 higher statistical probability of retained roots occurrence than the control group (*p* = 0.005) (Figure 2).

No statistically significant differences were observed while analyzing the number of impacted teeth. In the examined group, impacted teeth were noted in 36 patients (none were observed in 50 people). There were 56 patients with impacted teeth in the control group (50 were not diagnosed with that problem).

**Figure 3:**
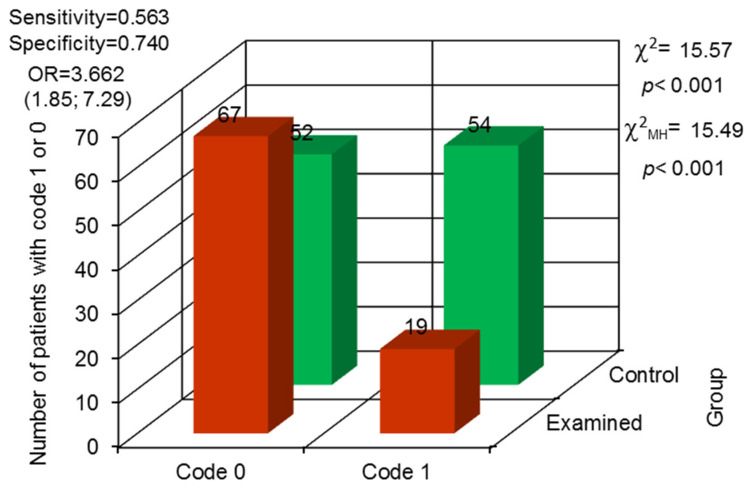
Odds ratio (OR) with 95% confidence interval and assessment for the variability in differences between both groups concerning endodontically treated teeth (code 0—not observed, code 1—observed).

The endodontic treatment needs were significantly higher in the examined group (*p* = 0.015). Endodontic treatment in prisoners was observed significantly less often (*p* < 0.001). In the examined group, it was confirmed that 19 prisoners had root canal treatment performed, and no endodontic treatment was performed in 67 prisoners. In the control group, 54 patients had endodontically treated teeth and in 52 that procedure was not noted. The odds ratio proved that prisoners were characterized by 3.6 times higher chance of no root canal treatment presence than the control group (Figure 3).

**Figure 4:**
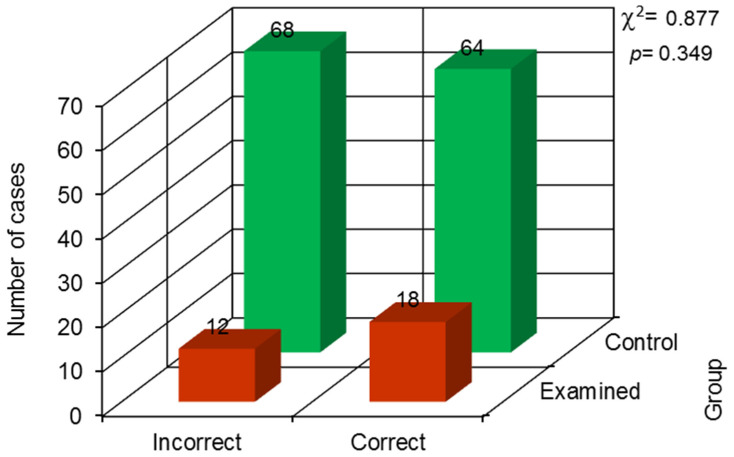
The results of the chi-square independence test for the De Cleen index.

**Figure 5:**
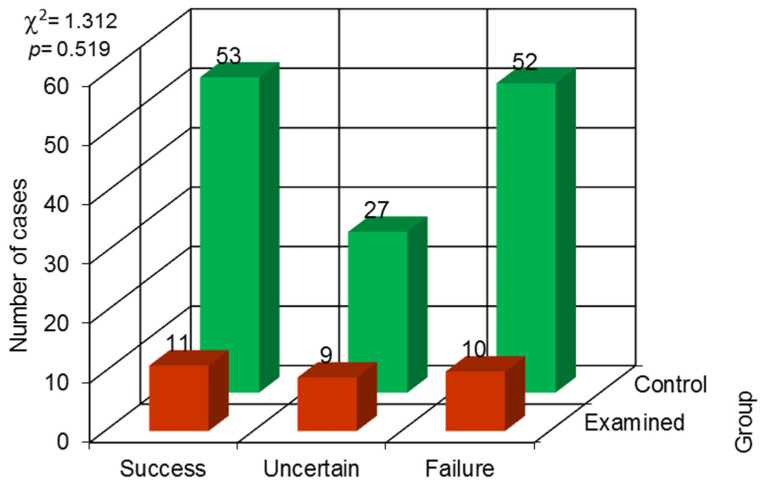
The results of the chi-square independence test for the Strindberg index.

No statistically significant difference was observed in the quality of root canal treatment assessed with the De Cleena and Strindberg indices (result of the chi-square independence test) (Figure 4 and Figure 5).

**Figure 6:**
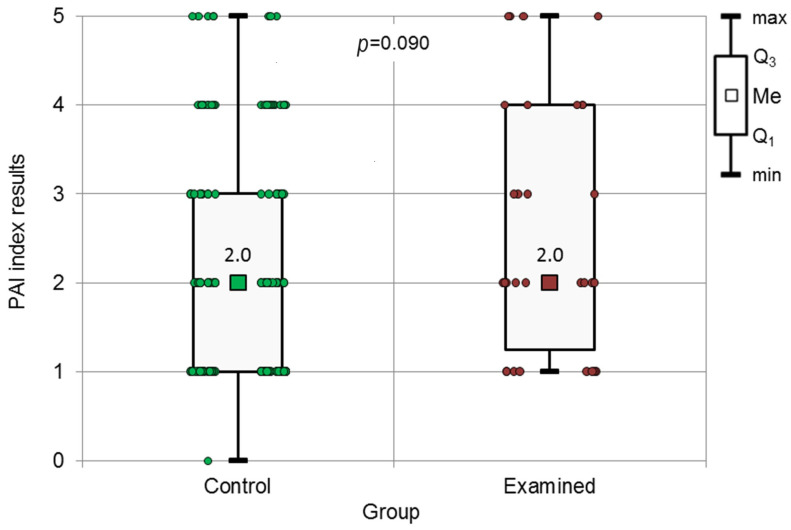
The results of the Mann–Whitney test applied to the PAI index, for both groups.

No statistically significant difference was observed in the PAI index between the examined and control groups (Figure 6).

The results of the chi-square independence test proved no statistically significant difference between the two groups in the presence of the symptoms of improper root canal obturation, such as excessive obturating material observed in periapical tissues and lack of homogeneity (Bołtacz–Rzepkowska modification). 

**Figure 7:**
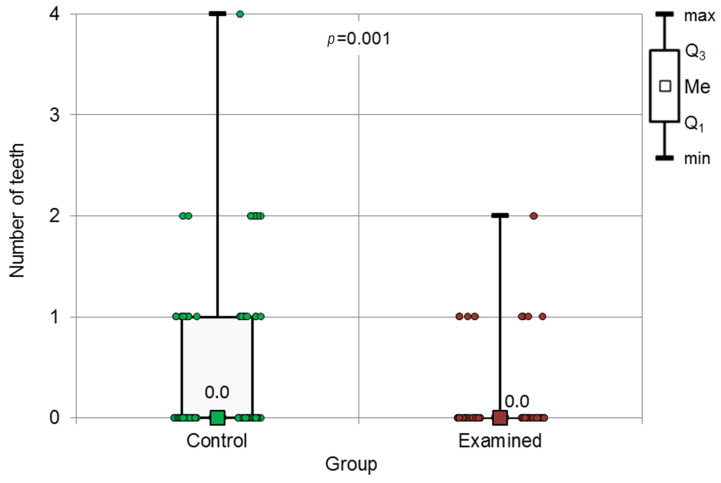
The results of the Mann–Whitney test concerning the number of teeth needing reendow treatment, for both groups.

There was no statistically significant difference between the groups in the presence of periapical changes in endodontically treated teeth (*p* = 0.389). In the control group, in which the frequency of the control appointments in the dental office was significantly higher, the decision on the reendo treatment was also significantly more frequent. The number of the teeth that qualified as those needing endodontical treatment for the second time was significantly higher in the control group (*p* = 0.001) (Figure 7).

**Figure 8:**
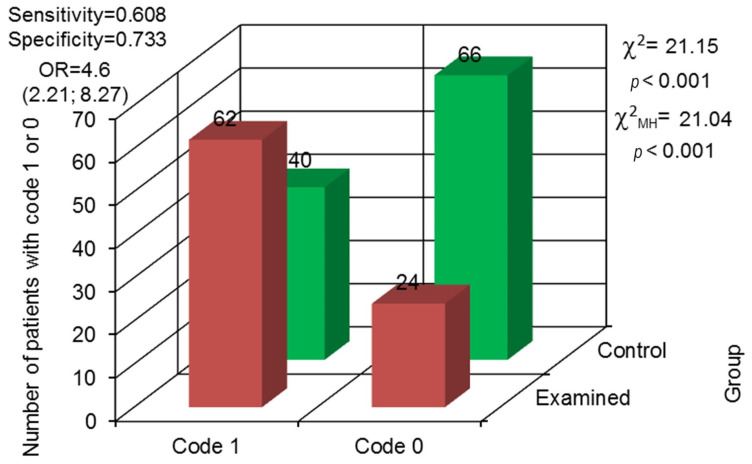
Odds ratio (OR) with 95% confidence interval and assessment for the variability in differences between both groups concerning the occurrence of teeth with no endodontic treatment and periapical lesions (code 0—not observed, code 1—observed).

Periapical changes in teeth with no previous endodontic treatment were also observed, more often in the examined group. In that group, it was observed in 62 prisoners and not noted in 24, while in the control group, periapical changes were observed in 40 patients and were not in 66. The odds ratio proved that the examined group was characterized by over 4.2 times higher probability for periapical changes occurrence in teeth with no previous endodontic treatment, when compared to the control group (*p* < 0.001) (Figure 8).

**Figure 9:**
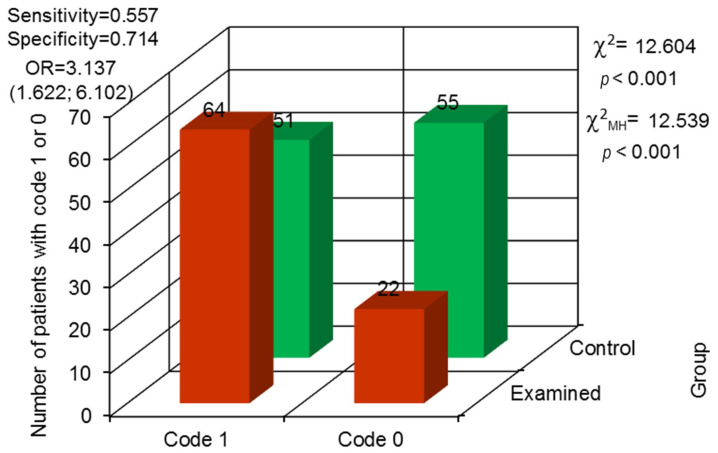
Odds ratio (OR) with 95% confidence interval and assessment for the variability in differences between both groups concerning horizontal bone atrophy (code 0—not observed, code 1—observed).

To assess the condition of the alveolar process in both maxilla and mandible, horizontal atrophy was analyzed. It was observed significantly more often in the examined group, present in 64 prisoners and not present in 22. In the control group, horizontal atrophy was diagnosed in 51 people and lacking in 55. The odds ratio proved that the probability for horizontal atrophy in the alveolar processes in maxilla and mandible occurrence in the examined group is over three times higher than in the control group (*p* < 0.001) (Figure 9). 

**Figure 10:**
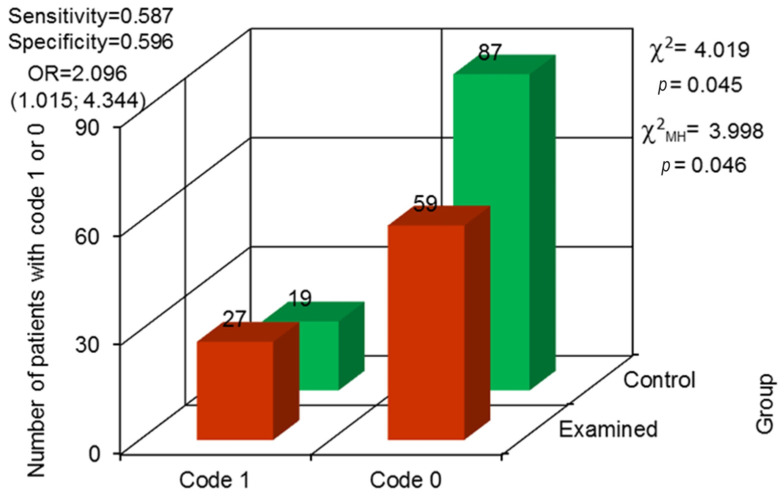
Odds ratio (OR) with 95% confidence interval and assessment for the variability in differences between both groups concerning vertical bone atrophy (code 0—not observed, code 1—observed).

The differences in the results for vertical atrophy of the alveolar process showed a tendency toward statistical significance (*p* = 0.045) (Figure 10). It was observed more often in the examined group—present in 27 prisoners and not present in 19. In the control group, vertical atrophy was diagnosed in 59 people and lacking in 87. 

## 4. Discussion

Analysis of the available literature on dental care and the stomatognathic system in prisoners was one of the completed aims of our study. Only one article compared the oral status of prisoners with a control group composed of people who were not sentenced to imprisonment [3]. Concerning hard tissues: the percentage of prisoners with decayed (D) (90.2%) and missing teeth (M) (80.5%) was higher than in the control group (D = 57%, M = 6 0.8%); the percentage of prisoners with filled teeth (F) was 31.7%, while in the control it was 50.6%; the median DMF was higher in prisoners than in the control group (M = 8 v M = %, *p* = 0.001). One article, similar to ours, presented results of the medical history analysis. The analysis of the medical history was based on the *International Classification of Diseases 10th Revision*—Clinical Modification (ICD-10CM) and proved that the percentage prevalence of chosen diseases was as follows: caries—92.4%, excessive teeth attrition—56.4%, calculus—89.5%, pulp and periapical tissue diseases—87.9%, gingivitis—33.1%, periodontitis—55.7%, gingival recession—26.9%, cheilitis—64.9%, cheek and lip biting—53.1%, leukoplakia—8.2%, leukokeratosis nicotina palati—28.8%, hyperplasia of mucosa—6.8%, tongue diseases—21.9%, complete loss of teeth—7.5%, partial loss of teeth—81.9%, and candidiasis—4.9%. Performing the retrospective analysis and usage of the control group made it possible to compare the results for particular parameters (provided that there is no significant difference in age between the examined and control groups). Each chosen article discussed the DMFT index and its component values. The value for F was significantly higher in the control group (*p* < 0.001), 5.5, in comparison to the examined group, 2.0. It confirmed the statement that prisoners were characterized by higher dental treatment needs and lower treatment realization. The low proportion of filled teeth among prisoners could be attributed to the difficulty in accessing dental services, their negative attitude toward dental health, and limited resources available in prison settings [5]. The total DMF index value in our examined group was 17.50 and 16.00 in the control group; no statistically significant differences were observed (*p* = 0.207) (Mann–Whitney test). In another publication, the mean DMFT index value found among inmates was quite high (19.72), similar to that of prisoners in Australia (20.4), United Kingdom (14.35), and South Africa (15.45). Dissimilar results were found in studies from India (5.26), Western Africa (6.5), and Italy (9.8), which showed that caries experience varied considerably within prison populations in different countries, and even within a country [4]. We observed that D and M values were significantly higher in the examined group (*p* < 0.001); the values were D—7.0 in the examined group and 5.0 in the control group, and M was 5.0 in the examined group and 2.5 in the control group. This finding is in agreement with studies carried out by Veera Reddy, who found that 57.1% of prisoners in Karnataka jails had one or more missing (M) teeth due to the lack of dental services [2]. Surgical treatment was also neglected. The odds ratio proved that the examined group was characterized by almost 2.5 higher statistical probability of retained roots occurrence than the control group (*p* = 0.005). The prevalence of dental caries was found to be 92.5% with a DMFT value of 5.26. The same studies reported higher values of DMFT [2,6,7,9]. Clare observed that there was a substantial reduction in dental caries among prisoners who had been in prison continuously for 3 years. The mean number of decayed surfaces was reduced from 6.7 to 3.6 (46.3% reduction). This resulted from the restoration of decayed teeth, extraction of hopeless teeth, and the availability and utilization of dental health services in prison [11].

The analysis of the orthopantomograms enabled the holistic assessment not only of the surgical treatment needs, but also of the quality and needs for the endodontic treatment. This type of analysis is not widely discussed in the literature. Kondratyev et al. noted that dental care was only searched out when pain became intolerable. Inmates with symptomatic endodontic diseases usually searched for dental treatment. However, they often manifested their willingness for dental extraction instead of multiple visit endodontic treatment [7]. Our own research proved that there was no statistically significant difference between the groups in the presence of periapical changes in endodontically treated teeth (*p* = 0.389). This could lead to the conclusion that the implemented treatment was of comparable effectiveness for both prisoners and the control group. But health behaviors and the educational status of Polish and, for instance, Finnish prisoners, were considerably poorer than those of the general population [6]. In the control group, in which the frequency of the control appointments in the dental office was significantly higher, the decision on the reendo treatment was also significantly more frequent. High quality, careful, and frequent dental check-ups can resulted in revealing particular needs for treatment. The number of the teeth qualified as those that need to be endodontically treated for the second time was significantly higher in the control group (*p* = 0.001). The odds ratio proved that the examined group was characterized by over 4.2 times higher probability for periapical changes occurrence in teeth with no previous endodontic treatment, when compared to the control group (*p* < 0.001). 

To assess the condition of the alveolar process for both maxilla and mandible, horizontal atrophy was analyzed. It was observed significantly more often in the examined group—present in 64 prisoners and not present in 22. Observations on the occurrence of horizontal atrophy correlated with the condition of the oral cavity in prisoners, which was assessed with the DMF index and found to be worse than in the control group [2,6,7,9,14,15]. In the control group, horizontal atrophy was diagnosed in 51 people and lacking in 55. The odds ratio proved that the probability for the occurrence of horizontal atrophy in maxilla and mandible alveolar processes in the examined group was over three times higher than in the control group (*p* < 0.001). 

The differences in the results for vertical atrophy in the alveolar process showed the tendency toward statistical significance. It was observed more often in the examined group—present in 27 prisoners and not present in 59. In the control group, vertical atrophy was diagnosed in 59 people and lacking in 87. The observed differences in both groups were probably a result of a higher number of teeth that underwent more complicated treatment (i.e., endodontic) in the control group. These types of dental procedures might be followed by local atrophy of the bone [14,15].

### Limitations of the Study

The authors must admit that the study conducted had a few limitations. The most important one seemed to be the fact that no patient was examined, but only analysis of the available medical history was performed. The opportunity for examination could enrich the results with more information, for example, the social status of the participants or any other that result from direct conversation with the patient.

Undoubtedly, more complex information on the oral status of the prisoners could be gained if there was a possibility for analysis of their medical history from the period before their incarceration. 

We also need to admit that the best method to assess the outcome of endodontic procedures is through computed tomography scans rather than panoramic X-rays.

## 5. Conclusions

The oral status of inmates is worse when compared to those who live in freedom. That is why there is a need to prepare a scheme to improve the condition of the stomatognathic system in prisoners. 

Undoubtedly, the research conducted on the inmates is the source of much more detailed data; however, the retrospective analysis of the medical history provides a chance to analyze the particular stages in the treatment and to analyze the available X-rays (enriches the results with the condition of the periapical tissues). 

## Data Availability

The data presented in the study are available on request form the corresponding author.
